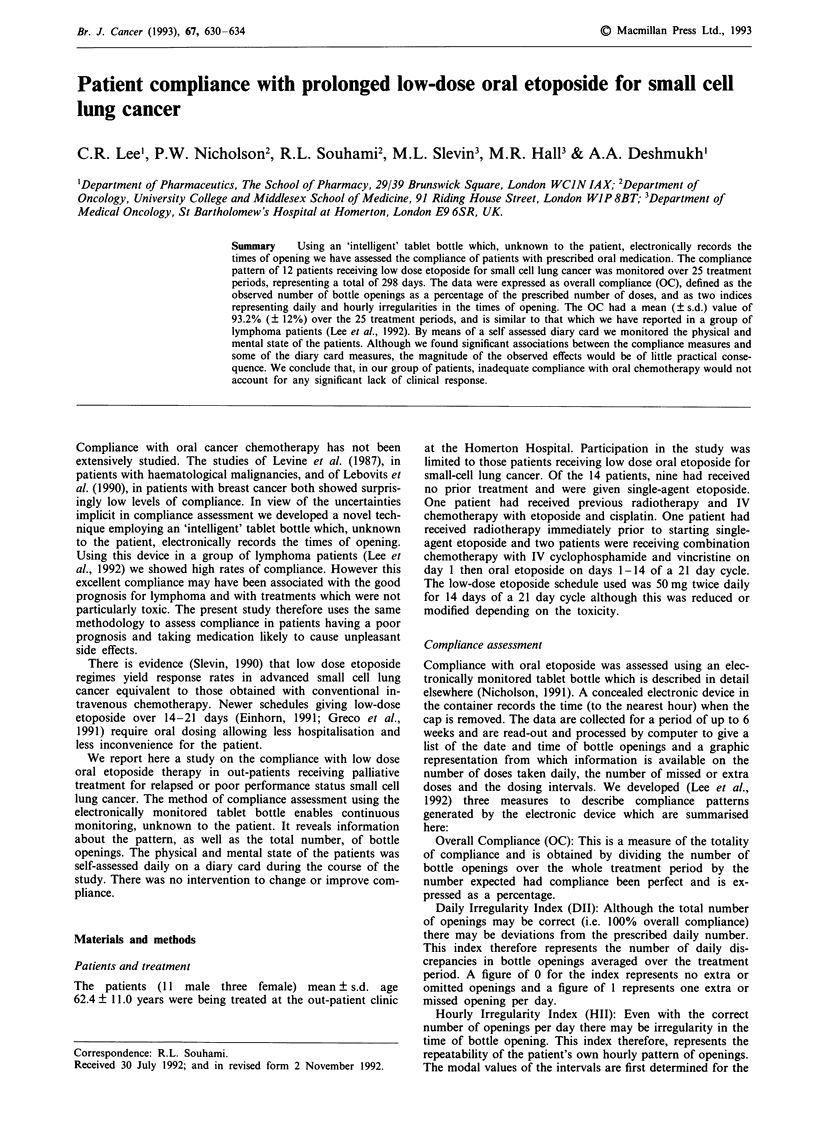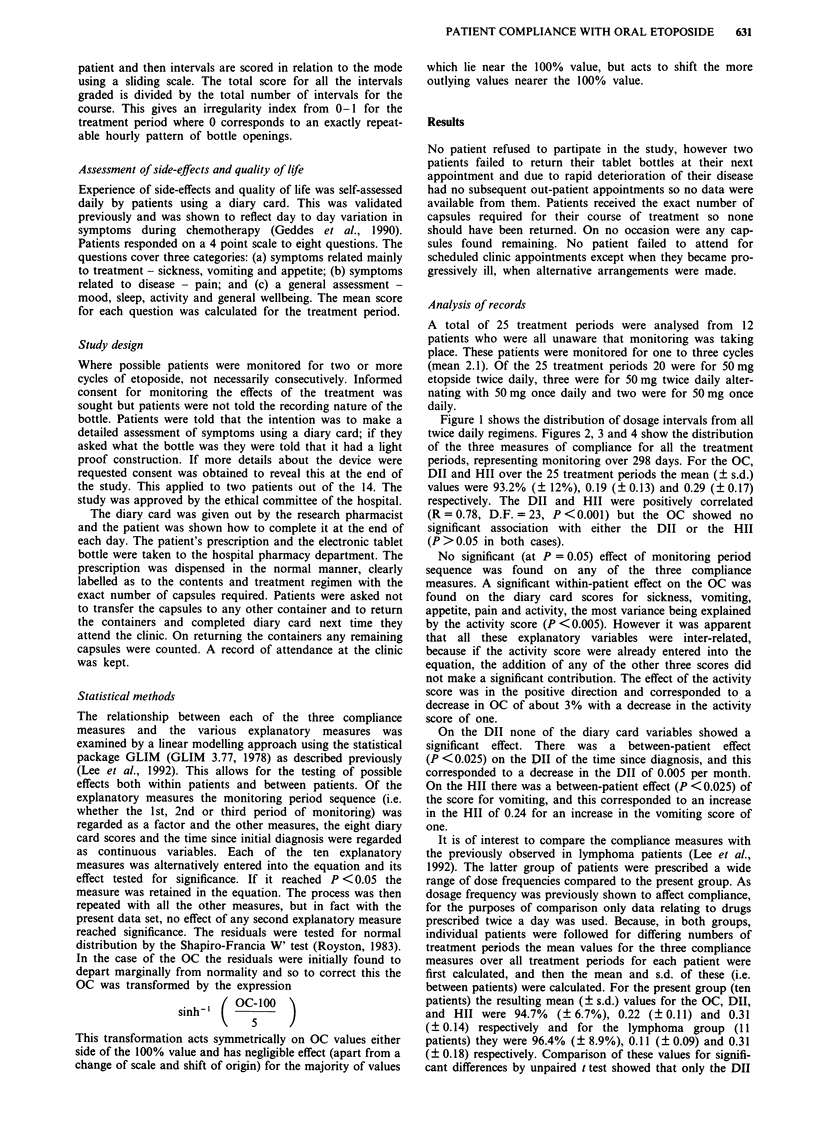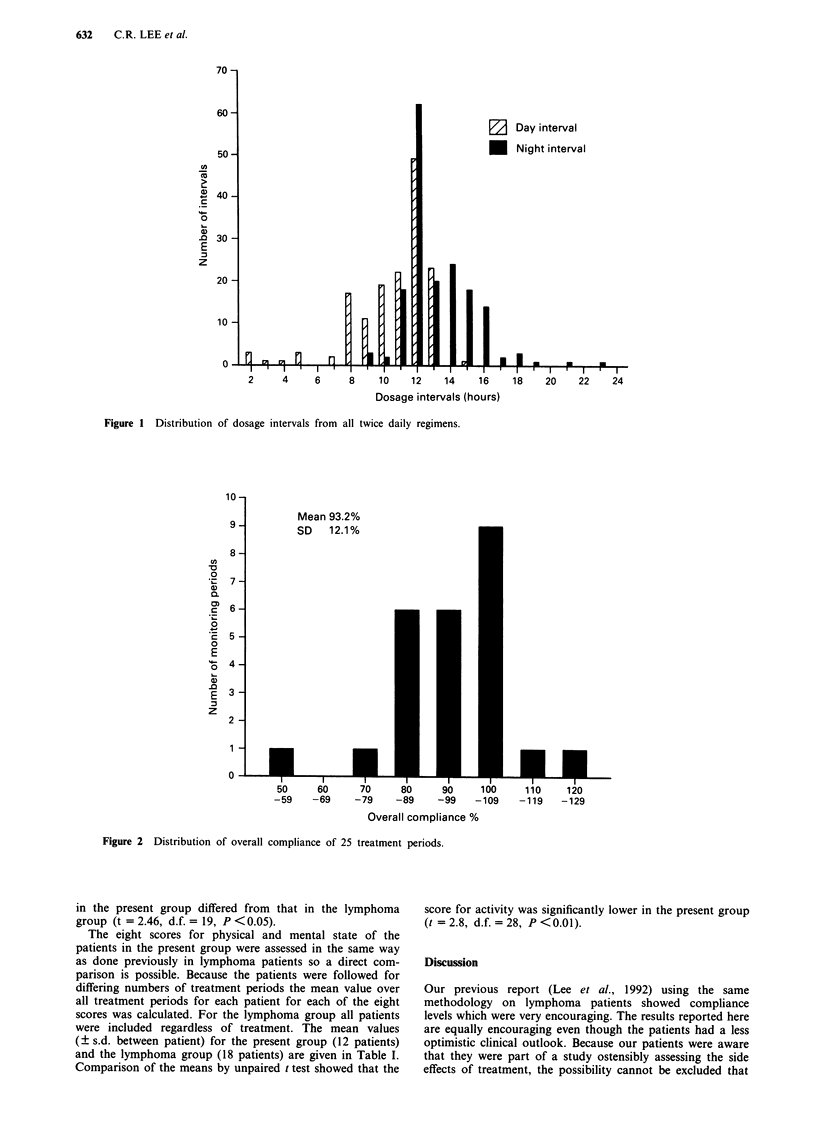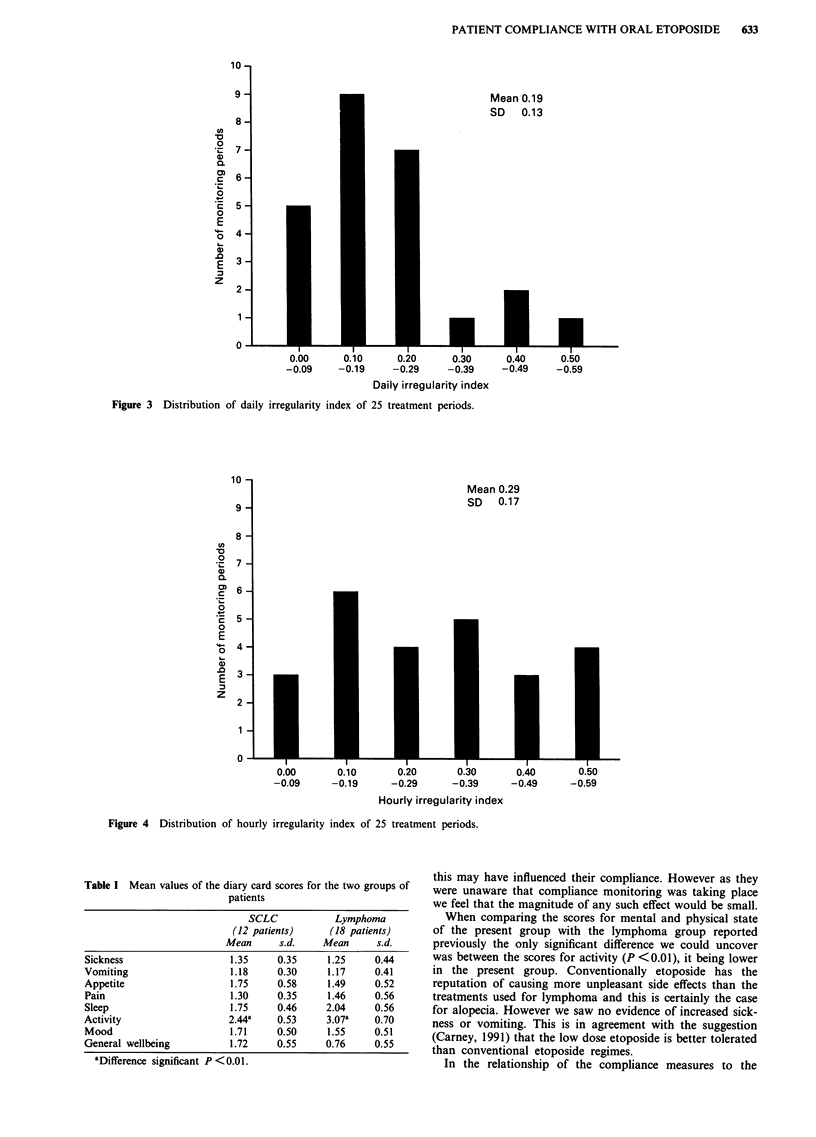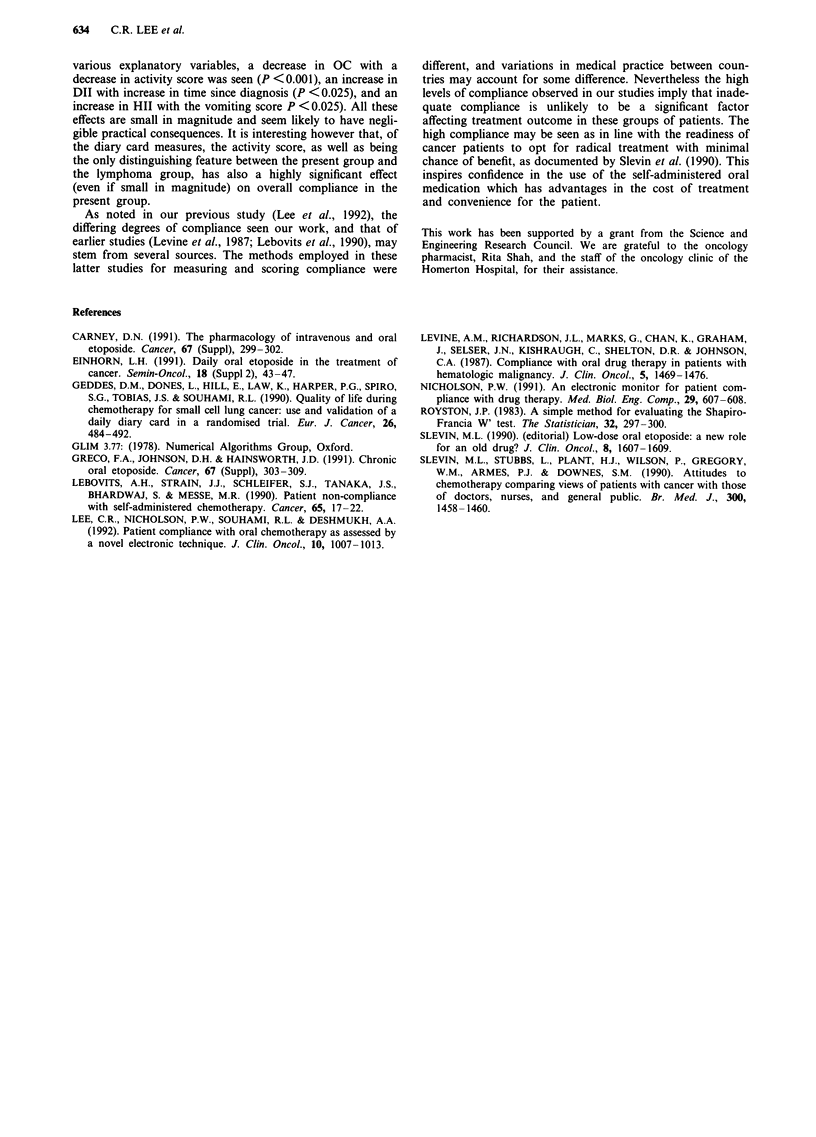# Patient compliance with prolonged low-dose oral etoposide for small cell lung cancer.

**DOI:** 10.1038/bjc.1993.115

**Published:** 1993-03

**Authors:** C. R. Lee, P. W. Nicholson, R. L. Souhami, M. L. Slevin, M. R. Hall, A. A. Deshmukh

**Affiliations:** Department of Pharmaceutics, School of Pharmacy, London, UK.

## Abstract

Using an 'intelligent' tablet bottle which, unknown to the patient, electronically records the times of opening we have assessed the compliance of patients with prescribed oral medication. The compliance pattern of 12 patients receiving low dose etoposide for small cell lung cancer was monitored over 25 treatment periods, representing a total of 298 days. The data were expressed as overall compliance (OC), defined as the observed number of bottle openings as a percentage of the prescribed number of doses, and as two indices representing daily and hourly irregularities in the times of opening. The OC had a mean (+/- s.d.) value of 93.2% (+/- 12%) over the 25 treatment periods, and is similar to that which we have reported in a group of lymphoma patients (Lee et al., 1992). By means of a self assessed diary card we monitored the physical and mental state of the patients. Although we found significant associations between the compliance measures and some of the diary card measures, the magnitude of the observed effects would be of little practical consequence. We conclude that, in our group of patients, inadequate compliance with oral chemotherapy would not account for any significant lack of clinical response.


Br. J. Cancer (1993), 67, 630-634 © Macmillan Press Ltd., 1993

### Patient compliance with prolonged low-dose oral etoposide for small cell lung cancer

C.R. Lee[1], P.W. Nicholson[2], R.L. Souhami[2], M.L. Slevin[3], M.R. Hall[3] & A.A. Deshmukh[1]

[1]*Department of Pharmaceutics, The School of Pharmacy, 29/39 Brunswick Square, London WC1N 1AX;* [2]*Department of Oncology, University College and Middlesex School of Medicine, 91 Riding House Street, London W1P 8BT;* [3]*Department of Medical Oncology, St Bartholomew's Hospital at Homerton, London E9 6SR, UK.*

**Summary** Using an 'intelligent' tablet bottle which, unknown to the patient, electronically records the times of opening we have assessed the compliance of patients with prescribed oral medication. The compliance pattern of 12 patients receiving low dose etoposide for small cell lung cancer was monitored over 25 treatment periods, representing a total of 298 days. The data were expressed as overall compliance (OC), defined as the observed number of bottle openings as a percentage of the prescribed number of doses, and as two indices representing daily and hourly irregularities in the times of opening. The OC had a mean (± s.d.) value of 93.2% (± 12%) over the 25 treatment periods, and is similar to that which we have reported in a group of lymphoma patients (Lee *et al.*, 1992). By means of a self assessed diary card we monitored the physical and mental state of the patients. Although we found significant associations between the compliance measures and some of the diary card measures, the magnitude of the observed effects would be of little practical consequence. We conclude that, in our group of patients, inadequate compliance with oral chemotherapy would not account for any significant lack of clinical response.

Compliance with oral cancer chemotherapy has not been extensively studied. The studies of Levine *et al.* (1987), in patients with haematological malignancies, and of Lebovits *et al.* (1990), in patients with breast cancer both showed surprisingly low levels of compliance. In view of the uncertainties implicit in compliance assessment we developed a novel technique employing an 'intelligent' tablet bottle which, unknown to the patient, electronically records the times of opening. Using this device in a group of lymphoma patients (Lee *et al.*, 1992) we showed high rates of compliance. However this excellent compliance may have been associated with the good prognosis for lymphoma and with treatments which were not particularly toxic. The present study therefore uses the same methodology to assess compliance in patients having a poor prognosis and taking medication likely to cause unpleasant side effects.

There is evidence (Slevin, 1990) that low dose etoposide regimes yield response rates in advanced small cell lung cancer equivalent to those obtained with conventional intravenous chemotherapy. Newer schedules giving low-dose etoposide over 14-21 days (Einhorn, 1991; Greco *et al.*, 1991) require oral dosing allowing less hospitalisation and less inconvenience for the patient.

We report here a study on the compliance with low dose oral etoposide therapy in out-patients receiving palliative treatment for relapsed or poor performance status small cell lung cancer. The method of compliance assessment using the electronically monitored tablet bottle enables continuous monitoring, unknown to the patient. It reveals information about the pattern, as well as the total number, of bottle openings. The physical and mental state of the patients was self-assessed daily on a diary card during the course of the study. There was no intervention to change or improve compliance.

### Materials and methods

### *Patients and treatment*

The patients (11 male three female) mean ± s.d. age 62.4 ± 11.0 years were being treated at the out-patient clinic at the Homerton Hospital. Participation in the study was limited to those patients receiving low dose oral etoposide for small-cell lung cancer. Of the 14 patients, nine had received no prior treatment and were given single-agent etoposide. One patient had received previous radiotherapy and IV chemotherapy with etoposide and cisplatin. One patient had received radiotherapy immediately prior to starting single-agent etoposide and two patients were receiving combination chemotherapy with IV cyclophosphamide and vincristine on day 1 then oral etoposide on days 1-14 of a 21 day cycle. The low-dose etoposide schedule used was 50 mg twice daily for 14 days of a 21 day cycle although this was reduced or modified depending on the toxicity.

### *Compliance assessment*

Compliance with oral etoposide was assessed using an electronically monitored tablet bottle which is described in detail elsewhere (Nicholson, 1991). A concealed electronic device in the container records the time (to the nearest hour) when the cap is removed. The data are collected for a period of up to 6 weeks and are read-out and processed by computer to give a list of the date and time of bottle openings and a graphic representation from which information is available on the number of doses taken daily, the number of missed or extra doses and the dosing intervals. We developed (Lee *et al.*, 1992) three measures to describe compliance patterns generated by the electronic device which are summarised here:

Overall Compliance (OC): This is a measure of the totality of compliance and is obtained by dividing the number of bottle openings over the whole treatment period by the number expected had compliance been perfect and is expressed as a percentage.

Daily Irregularity Index (DII): Although the total number of openings may be correct (i.e. 100% overall compliance) there may be deviations from the prescribed daily number. This index therefore represents the number of daily discrepancies in bottle openings averaged over the treatment period. A figure of 0 for the index represents no extra or omitted openings and a figure of 1 represents one extra or missed opening per day.

Hourly Irregularity Index (HII): Even with the correct number of openings per day there may be irregularity in the time of bottle opening. This index therefore, represents the repeatability of the patient's own hourly pattern of openings. The modal values of the intervals are first determined for the

Correspondence: R.L. Souhami.
Received 30 July 1992; and in revised form 2 November 1992.

patient and then intervals are scored in relation to the mode using a sliding scale. The total score for all the intervals graded is divided by the total number of intervals for the course. This gives an irregularity index from 0–1 for the treatment period where 0 corresponds to an exactly repeatable hourly pattern of bottle openings.

*Assessment of side-effects and quality of life*

Experience of side-effects and quality of life was self-assessed daily by patients using a diary card. This was validated previously and was shown to reflect day to day variation in symptoms during chemotherapy (Geddes *et al.*, 1990). Patients responded on a 4 point scale to eight questions. The questions cover three categories: (a) symptoms related mainly to treatment – sickness, vomiting and appetite; (b) symptoms related to disease – pain; and (c) a general assessment – mood, sleep, activity and general wellbeing. The mean score for each question was calculated for the treatment period.

*Study design*

Where possible patients were monitored for two or more cycles of etoposide, not necessarily consecutively. Informed consent for monitoring the effects of the treatment was sought but patients were not told the recording nature of the bottle. Patients were told that the intention was to make a detailed assessment of symptoms using a diary card; if they asked what the bottle was they were told that it had a light proof construction. If more details about the device were requested consent was obtained to reveal this at the end of the study. This applied to two patients out of the 14. The study was approved by the ethical committee of the hospital.

The diary card was given out by the research pharmacist and the patient was shown how to complete it at the end of each day. The patient's prescription and the electronic tablet bottle were taken to the hospital pharmacy department. The prescription was dispensed in the normal manner, clearly labelled as to the contents and treatment regimen with the exact number of capsules required. Patients were asked not to transfer the capsules to any other container and to return the containers and completed diary card next time they attend the clinic. On returning the containers any remaining capsules were counted. A record of attendance at the clinic was kept.

*Statistical methods*

The relationship between each of the three compliance measures and the various explanatory measures was examined by a linear modelling approach using the statistical package GLIM (GLIM 3.77, 1978) as described previously (Lee *et al.*, 1992). This allows for the testing of possible effects both within patients and between patients. Of the explanatory measures the monitoring period sequence (i.e. whether the 1st, 2nd or third period of monitoring) was regarded as a factor and the other measures, the eight diary card scores and the time since initial diagnosis were regarded as continuous variables. Each of the ten explanatory measures was alternatively entered into the equation and its effect tested for significance. If it reached $P < 0.05$ the measure was retained in the equation. The process was then repeated with all the other measures, but in fact with the present data set, no effect of any second explanatory measure reached significance. The residuals were tested for normal distribution by the Shapiro-Francia W' test (Royston, 1983). In the case of the OC the residuals were initially found to depart marginally from normality and so to correct this the OC was transformed by the expression

$$\sinh^{-1}\left(\frac{\text{OC-100}}{5}\right)$$

This transformation acts symmetrically on OC values either side of the 100% value and has negligible effect (apart from a change of scale and shift of origin) for the majority of values which lie near the 100% value, but acts to shift the more outlying values nearer the 100% value.

### Results

No patient refused to partipate in the study, however two patients failed to return their tablet bottles at their next appointment and due to rapid deterioration of their disease had no subsequent out-patient appointments so no data were available from them. Patients received the exact number of capsules required for their course of treatment so none should have been returned. On no occasion were any capsules found remaining. No patient failed to attend for scheduled clinic appointments except when they became progressively ill, when alternative arrangements were made.

*Analysis of records*

A total of 25 treatment periods were analysed from 12 patients who were all unaware that monitoring was taking place. These patients were monitored for one to three cycles (mean 2.1). Of the 25 treatment periods 20 were for 50 mg etopside twice daily, three were for 50 mg twice daily alternating with 50 mg once daily and two were for 50 mg once daily.

Figure 1 shows the distribution of dosage intervals from all twice daily regimens. Figures 2, 3 and 4 show the distribution of the three measures of compliance for all the treatment periods, representing monitoring over 298 days. For the OC, DII and HII over the 25 treatment periods the mean (± s.d.) values were 93.2% (± 12%), 0.19 (± 0.13) and 0.29 (± 0.17) respectively. The DII and HII were positively correlated ($R = 0.78$, $D.F. = 23$, $P < 0.001$) but the OC showed no significant association with either the DII or the HII ($P > 0.05$ in both cases).

No significant (at $P = 0.05$) effect of monitoring period sequence was found on any of the three compliance measures. A significant within-patient effect on the OC was found on the diary card scores for sickness, vomiting, appetite, pain and activity, the most variance being explained by the activity score ($P < 0.005$). However it was apparent that all these explanatory variables were inter-related, because if the activity score were already entered into the equation, the addition of any of the other three scores did not make a significant contribution. The effect of the activity score was in the positive direction and corresponded to a decrease in OC of about 3% with a decrease in the activity score of one.

On the DII none of the diary card variables showed a significant effect. There was a between-patient effect ($P < 0.025$) on the DII of the time since diagnosis, and this corresponded to a decrease in the DII of 0.005 per month. On the HII there was a between-patient effect ($P < 0.025$) of the score for vomiting, and this corresponded to an increase in the HII of 0.24 for an increase in the vomiting score of one.

It is of interest to compare the compliance measures with the previously observed in lymphoma patients (Lee *et al.*, 1992). The latter group of patients were prescribed a wide range of dose frequencies compared to the present group. As dosage frequency was previously shown to affect compliance, for the purposes of comparison only data relating to drugs prescribed twice a day was used. Because, in both groups, individual patients were followed for differing numbers of treatment periods the mean values for the three compliance measures over all treatment periods for each patient were first calculated, and then the mean and s.d. of these (i.e. between patients) were calculated. For the present group (ten patients) the resulting mean (± s.d.) values for the OC, DII, and HII were 94.7% (± 6.7%), 0.22 (± 0.11) and 0.31 (± 0.14) respectively and for the lymphoma group (11 patients) they were 96.4% (± 8.9%), 0.11 (± 0.09) and 0.31 (± 0.18) respectively. Comparison of these values for significant differences by unpaired *t* test showed that only the DII

**Figure 1** Distribution of dosage intervals from all twice daily regimens.

**Figure 2** Distribution of overall compliance of 25 treatment periods.

in the present group differed from that in the lymphoma group (t = 2.46, d.f. = 19, $P < 0.05$).

The eight scores for physical and mental state of the patients in the present group were assessed in the same way as done previously in lymphoma patients so a direct comparison is possible. Because the patients were followed for differing numbers of treatment periods the mean value over all treatment periods for each patient for each of the eight scores was calculated. For the lymphoma group all patients were included regardless of treatment. The mean values (± s.d. between patient) for the present group (12 patients) and the lymphoma group (18 patients) are given in Table I. Comparison of the means by unpaired $t$ test showed that the score for activity was significantly lower in the present group ($t$ = 2.8, d.f. = 28, $P < 0.01$).

### Discussion

Our previous report (Lee *et al.*, 1992) using the same methodology on lymphoma patients showed compliance levels which were very encouraging. The results reported here are equally encouraging even though the patients had a less optimistic clinical outlook. Because our patients were aware that they were part of a study ostensibly assessing the side effects of treatment, the possibility cannot be excluded that

**Figure 3** Distribution of daily irregularity index of 25 treatment periods.

**Figure 4** Distribution of hourly irregularity index of 25 treatment periods.

**Table I** Mean values of the diary card scores for the two groups of patients

| | *SCLC (12 patients)* | | *Lymphoma (18 patients)* | |
|---|---|---|---|---|
| | *Mean* | *s.d.* | *Mean* | *s.d.* |
| Sickness | 1.35 | 0.35 | 1.25 | 0.44 |
| Vomiting | 1.18 | 0.30 | 1.17 | 0.41 |
| Appetite | 1.75 | 0.58 | 1.49 | 0.52 |
| Pain | 1.30 | 0.35 | 1.46 | 0.56 |
| Sleep | 1.75 | 0.46 | 2.04 | 0.56 |
| Activity | 2.44[a] | 0.53 | 3.07[a] | 0.70 |
| Mood | 1.71 | 0.50 | 1.55 | 0.51 |
| General wellbeing | 1.72 | 0.55 | 0.76 | 0.55 |

[a]Difference significant $P < 0.01$.

this may have influenced their compliance. However as they were unaware that compliance monitoring was taking place we feel that the magnitude of any such effect would be small.

When comparing the scores for mental and physical state of the present group with the lymphoma group reported previously the only significant difference we could uncover was between the scores for activity ($P < 0.01$), it being lower in the present group. Conventionally etoposide has the reputation of causing more unpleasant side effects than the treatments used for lymphoma and this is certainly the case for alopecia. However we saw no evidence of increased sickness or vomiting. This is in agreement with the suggestion (Carney, 1991) that the low dose etoposide is better tolerated than conventional etoposide regimes.

In the relationship of the compliance measures to the various explanatory variables, a decrease in OC with a decrease in activity score was seen ($P < 0.001$), an increase in DII with increase in time since diagnosis ($P < 0.025$), and an increase in HII with the vomiting score $P < 0.025$). All these effects are small in magnitude and seem likely to have negligible practical consequences. It is interesting however that, of the diary card measures, the activity score, as well as being the only distinguishing feature between the present group and the lymphoma group, has also a highly significant effect (even if small in magnitude) on overall compliance in the present group.

As noted in our previous study (Lee *et al.*, 1992), the differing degrees of compliance seen our work, and that of earlier studies (Levine *et al.*, 1987; Lebovits *et al.*, 1990), may stem from several sources. The methods employed in these latter studies for measuring and scoring compliance were different, and variations in medical practice between countries may account for some difference. Nevertheless the high levels of compliance observed in our studies imply that inadequate compliance is unlikely to be a significant factor affecting treatment outcome in these groups of patients. The high compliance may be seen as in line with the readiness of cancer patients to opt for radical treatment with minimal chance of benefit, as documented by Slevin *et al.* (1990). This inspires confidence in the use of the self-administered oral medication which has advantages in the cost of treatment and convenience for the patient.

This work has been supported by a grant from the Science and Engineering Research Council. We are grateful to the oncology pharmacist, Rita Shah, and the staff of the oncology clinic of the Homerton Hospital, for their assistance.

### References

CARNEY, D.N. (1991). The pharmacology of intravenous and oral etoposide. *Cancer*, **67** (Suppl), 299–302.

EINHORN, L.H. (1991). Daily oral etoposide in the treatment of cancer. *Semin-Oncol.*, **18** (Suppl 2), 43–47.

GEDDES, D.M., DONES, L., HILL, E., LAW, K., HARPER, P.G., SPIRO, S.G., TOBIAS, J.S. & SOUHAMI, R.L. (1990). Quality of life during chemotherapy for small cell lung cancer: use and validation of a daily diary card in a randomised trial. *Eur. J. Cancer*, **26**, 484–492.

GLIM 3.77: (1978). Numerical Algorithms Group, Oxford.

GRECO, F.A., JOHNSON, D.H. & HAINSWORTH, J.D. (1991). Chronic oral etoposide. *Cancer*, **67** (Suppl), 303–309.

LEBOVITS, A.H., STRAIN, J.J., SCHLEIFER, S.J., TANAKA, J.S., BHARDWAJ, S. & MESSE, M.R. (1990). Patient non-compliance with self-administered chemotherapy. *Cancer*, **65**, 17–22.

LEE, C.R., NICHOLSON, P.W., SOUHAMI, R.L. & DESHMUKH, A.A. (1992). Patient compliance with oral chemotherapy as assessed by a novel electronic technique. *J. Clin. Oncol.*, **10**, 1007–1013.

LEVINE, A.M., RICHARDSON, J.L., MARKS, G., CHAN, K., GRAHAM, J., SELSER, J.N., KISHRAUGH, C., SHELTON, D.R. & JOHNSON, C.A. (1987). Compliance with oral drug therapy in patients with hematologic malignancy. *J. Clin. Oncol.*, **5**, 1469–1476.

NICHOLSON, P.W. (1991). An electronic monitor for patient compliance with drug therapy. *Med. Biol. Eng. Comp.*, **29**, 607–608.

ROYSTON, J.P. (1983). A simple method for evaluating the Shapiro-Francia W' test. *The Statistician*, **32**, 297–300.

SLEVIN, M.L. (1990). (editorial) Low-dose oral etoposide: a new role for an old drug? *J. Clin. Oncol.*, **8**, 1607–1609.

SLEVIN, M.L., STUBBS, L., PLANT, H.J., WILSON, P., GREGORY, W.M., ARMES, P.J. & DOWNES, S.M. (1990). Attitudes to chemotherapy comparing views of patients with cancer with those of doctors, nurses, and general public. *Br. Med. J.*, **300**, 1458–1460.